# Hearing higher-order Weyl exceptional rings in lossy metamaterials

**DOI:** 10.1093/nsr/nwag311

**Published:** 2026-05-30

**Authors:** Tao Liu, Franco Nori

**Affiliations:** School of Physics and Optoelectronics, South China University of Technology, China; RIKEN Center for Quantum Computing, Japan; Department of Physics, University of Michigan, USA

Topological physics integrates the mathematical concept of topology into the physics framework to characterize quantum phases of matter and explore their applications in materials science. In Hermitian regimes, topological semimetals with nontrivial bulk band crossings have attracted sustained interest [1]. For instance, Weyl semimetals feature Weyl degeneracy points in three-dimensional space, which carry topological charges characterized by Chern numbers and guarantee Fermi-arc surface states. Crucially, Weyl points may further exhibit higher-order topological properties protected via quantized bulk polarizations or multipole moments, thereby enabling the formation of topological hinge states.

Previous research on topological phases primarily addressed closed Hermitian systems. However, all realistic systems exhibit energy and matter exchange with their environment—a fundamental process introducing non-Hermitian effects [2]. As robust degenerate lines in three-dimensional non-Hermitian systems that do not require symmetry protection, exceptional lines can be realized through the introduction of non-Hermiticity via two schemes: (i) the direct splitting of nodal lines into exceptional lines [3], and (ii) the deformation of Weyl points into Weyl exceptional rings [4]. In particular, Weyl exceptional rings have stimulated significant experimental interest since their proposal [5], including the seminal realizations in photonic crystals [6] and synthetic phononic crystals [7].

Recently, Liu *et al.* proposed Weyl exceptional ring semimetals with higher-order topological properties [8]. Such higher-order Weyl exceptional ring semimetals are predicted to support Fermi-arc hinge states. However, owing to the complexity in practically implementing such non-Hermitian Hamiltonian, experimental research on higher-order Weyl exceptional rings progressed slowly despite the high theoretical promise. Very recently, Hu *et al.* developed an experimentally feasible model and presented the first realization of higher-order Weyl exceptional ring semimetals in a lossy acoustic metamaterial [9]. By introducing loss only at partial couplings, their scheme successfully overcomes the challenges in realizing such non-Hermitian topological semimetals.

**Figure 1.**
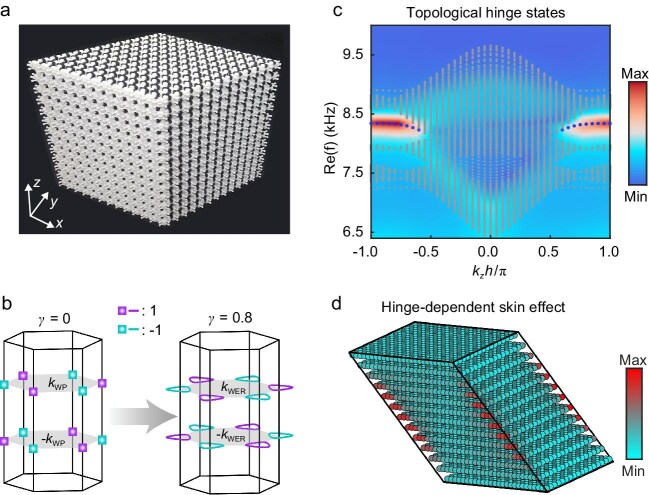
Higher-order Weyl exceptional ring semimetal in lossy acoustic metamaterials [9]. (a) Photograph of the acoustic metamaterial. (b) Distributions of the Weyl points (left panel) and Weyl exceptional rings (right panel) in the Brillouin zone. The topological charges are labeled as Chern number (+1) and (−1). (c) Measured hinge dispersion for the left hinge. The measured intensity is shown as a density map, the simulated result is denoted by dots, and the topological hinge states are indicated by the highlighted dispersion branch. (d) Distribution of Fermi-arc surface states in a tilted-prism acoustic metamaterial, showing hinge-dependent skin effect.

Based on a tight-binding model, the authors constructed the lossy acoustic metamaterial fabricated via 3D printing technology (Fig. 1a and b). The controllable loss was achieved by filling sponges into the wall of specific coupling tubes. They first investigated the first-order topological properties of Weyl exceptional rings, including Fermi-arc surface states and the surface-dependent skin effect of bulk states. Subsequently, they focused on two types of higher-order topological properties in the metamaterial. One is the topological hinge states protected by quantized bulk polarization, which exhibit purely real frequencies and enable robust acoustic propagation under loss (Fig. 1c). The other is a higher-order skin effect arising from the interplay between Fermi-arc surface states and the non-Hermitian skin effect, which possesses a hinge-dependent characteristic due to the surface mirror symmetry (Fig. 1d).

The work by Hu *et al.* successfully realizes the higher-order Weyl exceptional ring semimetals in acoustic metamaterials. Their design incorporates only real couplings and engineered loss, ensuring broad applicability across diverse systems and stimulating parallel studies.
